# Honokiol Improves Insulin Resistance, Hepatic Steatosis, and Inflammation in Type 2 Diabetic *db*/*db* Mice

**DOI:** 10.3390/ijms20092303

**Published:** 2019-05-09

**Authors:** Young-Je Kim, Un Ju Jung

**Affiliations:** 1 Department of Food Science and Nutrition, Kyungpook National University, Daegu 41566, Korea; breezy750@naver.com; 2 Department of Food Science and Nutrition, Pukyong National University, Busan 48513, Korea

**Keywords:** honokiol, type 2 diabetic *db*/*db* mice, anti-insulin resistance effect, anti-steatotic effect, anti-inflammatory effect

## Abstract

This study focuses on the effect of honokiol (HON) on glucose homeostasis, insulin resistance, dyslipidemia, hepatic steatosis, and inflammation in type 2 diabetic *db*/*db* mice. Male C57BL/KsJ-*db*/*db* mice were fed a normal diet with or without HON (0.02%, *w*/*w*) or pioglitazone (PIO, anti-diabetic agent, 0.01%, *w*/*w*) for 5 weeks. Blood biomarker, tissue morphology and enzymatic and genetic parameters were determined. PIO significantly decreased food intake, fasting blood glucose, and glycosylated hemoglobin (HbA1c) levels, but markedly increased body weight, adipose tissue weight, and plasma leptin levels. HON did not significantly affect food intake, body weight, or levels of plasma leptin and blood glucose. However, HON led to significant decreases in adipose tissue weight, plasma insulin, blood HbA1c and HOMA-IR levels and improved glucose tolerance. The anti-diabetic and anti-adiposity effects of HON were partially related to the inhibition of gluconeogenic enzymes and their mRNA expression in the liver; and the inhibition of lipogenic enzymes in adipose tissue, respectively. Unlike PIO, HON did not affect dyslipidemia, but ameliorated hepatic steatosis by inhibiting hepatic lipogenic enzymes activity. Moreover, HON exhibited anti-inflammatory effects similar to PIO. These results suggest that HON can protect against type 2 diabetes by improving insulin resistance, glucose and lipid metabolism, and inflammation.

## 1. Introduction

The incidence of type 2 diabetes has increased in recent years [1]. Worldwide, approximately 1 in 11 adults have diabetes, 90% of which have type 2 diabetes. Obesity is a well-established risk factor for type 2 diabetes, and insulin resistance is a key link between obesity and type 2 diabetes [2,3]. Although the mechanism by which obesity causes insulin resistance is unclear, chronic inflammation in white adipose tissue and increased adiposity are linked to local and systemic insulin resistance [4,5,6,7]. Nonalcoholic fatty liver disease (NAFLD), which is characterized by an increase in intrahepatic triglyceride content with or without inflammation and fibrosis, is common in people with type 2 diabetes, and is highly correlated with the development and severity of insulin resistance [8,9,10]. However, due to the complexity of obesity-related type 2 diabetes, the understanding of the pathogenic mechanisms that underlie the disease remain inadequate, and current therapeutics for improving type 2 diabetes are inefficient.

Honokiol (HON) is a biphenolic compound isolated from the bark and leaves of the Magnolia plant spp. It has been widely used in traditional medicine to treat thrombotic stroke, anxiety, and gastrointestinal symptoms in China and Japan. Many studies have demonstrated that HON confers a number of health benefits; it has cardioprotective, anti-microbial, anti-inflammatory, antiangiogenic, and anti-tumor properties [11,12,13,14,15]. In recent studies, HON protected pancreatic β-cells against high glucose and intermittent hypoxia-induced injury in type 2 diabetic rats [16] and ameliorated myocardial ischemia/reperfusion injury in type 1 diabetic rats by reducing oxidative stress and apoptosis [17]. Moreover, HON decreased fasting blood glucose levels and improved insulin sensitivity in high-fat diet- and streptozotocin-induced diabetic mice [18]. However, the mechanism by which HON exerts its anti-diabetic properties remains unclear, and no study has assessed the effect of HON on *db*/*db* mice. Leptin receptor deficient mice (*db*/*db* mice) are known to develop a phenotype that closely resembles human type 2 diabetes, with symptoms such as hyperphagia, obesity, hyperinsulinemia, insulin resistance, and NAFLD [19]. Hence, *db*/*db* mice are a useful model to determine the effects of HON on metabolic changes in type 2 diabetes. 

The aim of this study was to determine the effect of dietary HON on type 2 diabetes-related metabolic changes in *db*/*db* mice. The effects of HON were compared to those of pioglitazone (PIO), an anti-diabetic agent. We also investigated whether the effects of HON on *db*/*db* mice are related to the regulation of glucose and lipid metabolism, as well as inflammation in the liver and adipose tissue.

## 2. Results

### 2.1. Effect of HON on Food Intake, Body Weight, Adipose Tissue Weight, Plasma Leptin Levels, and Lipid-Regulating Enzyme Activity in Adipose Tissue 

**Figure 1:**
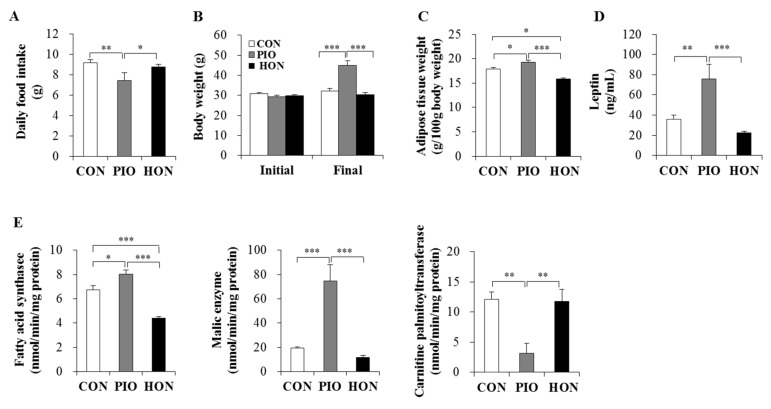
Effect of dietary HON on daily food intake (**A**), initial and final body weight (**B**), adipose tissue weight (**C**), plasma leptin levels (**D**), and the activity of lipid-regulating enzymes in adipose tissue (**E**) in *db*/*db* mice. Male C57BL/KsJ-*db*/*db* mice were fed a normal diet with or without HON (0.02%, *w*/*w*), or PIO (0.01%, *w*/*w*) for 5 weeks (*n* = 10 per group). Data are presented as mean ± SE. ^*^ *p* < 0.05, ^**^ *p* < 0.01, ^***^ *p* < 0.001. CON; control group, PIO; pioglitazone, HON; honokiol.

Supplementing the mouse diet with HON had no effect on daily food intake or the body weight in type 2 diabetic *db*/*db* mice, whereas PIO significantly increased the body weight and decreased the food intake of *db*/*db* mice compared to the CON group (Figure 1A,B). Moreover, treatment with PIO significantly increased adipose tissue weight compared to the CON group (Figure 1C). In contrast, the weight of adipose tissue in HON-treated mice was significantly lower than for both CON and PIO-treated mice. Plasma leptin levels also significantly increased in PIO-treated mice, whereas HON treatment decreased plasma leptin by 38% compared to the CON group (Figure 1D). 

We next examined the activity of enzymes that regulate lipid accumulation in adipose tissue. The activity of fatty acid synthase, the major biosynthetic enzyme in fatty acid synthesis, in adipose tissue was significantly lower in the HON-treated group relative to the CON group. HON treatment also tended to decrease the activity of another lipogenic malic enzyme in adipose tissue by 39% compared to the CON group (Figure 1E). In contrast, PIO led to a significant increase in both fatty acid synthase and malic enzyme activity in adipose tissue compared to the CON group (Figure 1E). The activity of carnitine palmitoyltransferase, an enzyme that catalyzes the rate-limiting step in the oxidation of long-chain fatty acids, was significantly lower in the adipose tissue of PIO-treated mice, whereas HON treatment resulted in no significant effects on adipose carnitine palmitoyltransferase activity compared to the CON group (Figure 1E).

### 2.2. Effect of HON on Glucose Homeostasis

**Figure 2:**
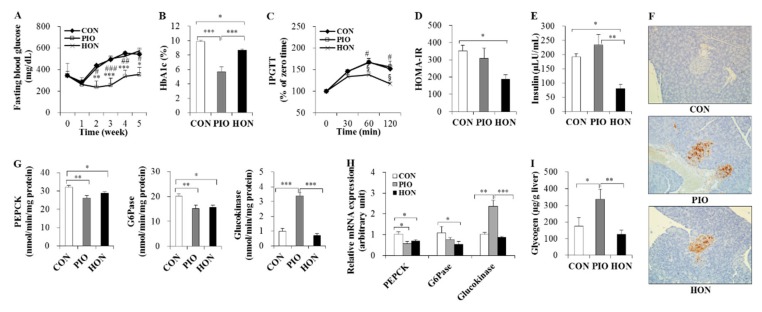
Effect of dietary HON on fasting blood glucose levels (**A**), blood HbA1c levels (**B**), IPGTT (**C**), HOMA-IR (**D**), plasma insulin levels (**E**), immunohistochemistry with anti-insulin (**F**), the activity and mRNA expression of hepatic glucose-regulating enzymes (**G**,**H**), and hepatic glycogen content (**I**) in *db*/*db* mice. Male C57BL/KsJ-*db*/*db* mice were fed a normal diet with or without HON (0.02%, *w*/*w*) or PIO (0.01%, *w*/*w*) for 5 weeks (*n* = 10 per group). (**A**,**C**): Data are presented as mean ± SE. CON vs. PIO ^*^ *p* < 0.05, ^**^ *p* < 0.01, ^***^ *p* < 0.001; PIO vs. HON ^#^ *p* < 0.05, ^##^ *p* < 0.01, ^###^ *p* < 0.001; CON vs. HON ^§^ *p* < 0.05. (**B**,**D**,**E**,**G**–**I**): Data are presented as mean ± SE. ^*^ *p* < 0.05, ^**^ *p* < 0.01, ^***^ *p* < 0.001. (**F**): Immunohistochemistry with antibodies against insulin. Original magnification ×200. CON; control group, PIO; pioglitazone, HON; honokiol, HbA1c; blood glycosylated hemoglobin, IPGTT; intraperitoneal glucose tolerance test, HOMA-IR; homeostatic index of insulin resistance, PEPCK; phosphoenolpyruvate carboxykinase, G6Pase; glucose-6-phosphatase.

As expected, fasting blood glucose and glycosylated hemoglobin (HbA1c) levels significantly decreased in response to PIO treatment in *db*/*db* mice (Figure 2A,B). HON did not significantly alter fasting blood glucose levels, but significantly decreased blood HbA1c levels compared to the CON group (Figure 2A,B). In the intraperitoneal glucose tolerance test (IPGTT) study, no significant differences in blood glucose levels at 30 min post glucose injection were observed among the three groups (Figure 2C). However, after 60 and 120 min, blood glucose levels remained significantly lower in the HON-treated group, compared to both the CON and PIO groups (Figure 2C). Homeostatic model assessment index of insulin resistance (HOMA-IR), a method used to assess β-cell function and insulin resistance using fasting glucose and insulin concentrations, also significantly decreased in response to HON treatment, compared to the CON group (Figure 2D). HON also resulted in a significant decrease in plasma insulin levels compared to both the CON and PIO groups (Figure 2E), and no significant differences in plasma glucagon levels were observed among the groups (data not shown). Immunostaining with an insulin antibody showed that *db*/*db* mice treated with HON or PIO exhibited strong staining compared to the CON group, suggesting the protection of β-cells by HON as well as PIO (Figure 2F).

To further examine the mechanism by which HON ameliorates glucose metabolism in *db*/*db* mice, we examined the mRNA expression and activity of enzymes that regulate glucose homeostasis in the liver. HON significantly decreased gluconeogenic phosphoenolpyruvate carboxykinase (PEPCK) and glucose-6-phosphatase (G6Pase) activity and mRNA expression in the liver relative to the CON group; however, hepatic glucokinase activity and mRNA expression were not significantly altered (Figure 2G,H). PIO led to a significant decrease in hepatic PEPCK and G6Pase activity in the liver compared to the CON group, whereas hepatic glucokinase activity significantly increased (Figure 2G). The changes in the mRNA levels of glucose metabolic enzymes in response to PIO were comparable to the enzyme activity in the liver (Figure 2H). Hepatic glycogen content also markedly increased in PIO-treated *db*/*db* mice compared to the CON mice, whereas HON did not significantly alter hepatic glycogen content (Figure 2I).

### 2.3. Effects of HON on Dyslipidemia and Hepatic Steatosis 

**Figure 3:**
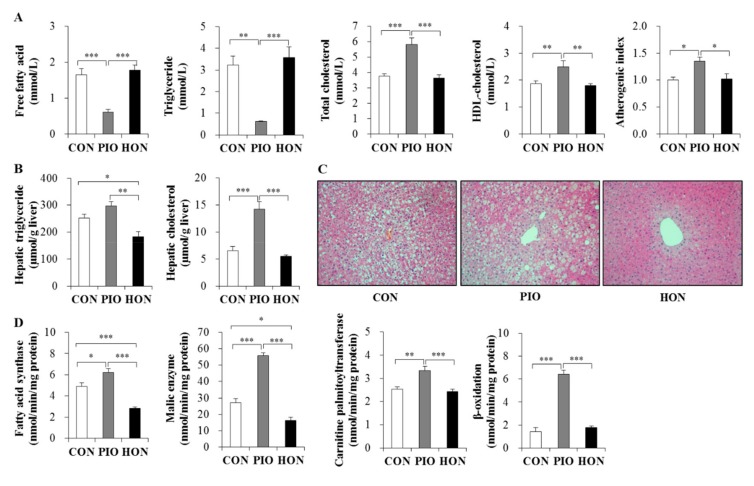
Effect of dietary HON on plasma lipid levels (**A**), hepatic lipid content (**B**), hepatic morphology (**C**), and hepatic lipid-regulating enzyme activity (**D**) in *db*/*db* mice. Male C57BL/KsJ-*db*/*db* mice were fed a normal diet with or without HON (0.02%, *w*/*w*) or PIO (0.01%, *w*/*w*) for 5 weeks (*n* = 10 per group). (**A**,**B**,**D**): Data are presented as mean ± SE. ^*^ *p* < 0.05, ^**^ *p* < 0.01, ^***^ *p* < 0.001. (**C**): Hepatic lipid droplet accumulation in fixed transverse liver sections stained with H&E. Original magnification ×200. CON; control group, PIO; pioglitazone, HON; honokiol. Atherogenic index = [(total cholesterol) − (HDL-cholesterol)]/(HDL-cholesterol).

Treatment with PIO significantly lowered the plasma free fatty acid and triglyceride levels in *db*/*db* mice, whereas no change was observed in HON-treated mice compared to CON mice (Figure 3A). In contrast, PIO significantly increased plasma total cholesterol levels and atherogenic index, and markedly increased plasma high-density lipoprotein (HDL)-cholesterol levels (Figure 3A). HON did not affect the levels of plasma total cholesterol, HDL-cholesterol, and atherogenic index (Figure 3A).

In contrast to the plasma free fatty acid and triglyceride levels, PIO significantly increased hepatic triglyceride content compared to the CON group (Figure 3B). PIO-treated mice had a significant increase in the hepatic cholesterol content compared to CON mice (Figure 3B). However, diet supplementation with HON led to a significant decrease in hepatic triglyceride content compared to the CON and PIO groups, although hepatic cholesterol content was not significantly altered by HON treatment (Figure 3B). Morphological analyses of the liver also indicated that lipid droplet accumulation was most pronounced in the PIO group, while treatment with HON resulted in a significant reduction in lipid droplet accumulation relative to the CON and PIO groups (Figure 3C).

Next, we determined the activity of enzymes regulating lipogenesis and fatty acid oxidation in the liver, in order to determine how HON is able to decrease hepatic lipid accumulation. Lipogenic fatty acid synthase and malic enzyme activity significantly increased in the livers of PIO-treated *db*/*db* mice, whereas treatment with HON led to a significant decrease in hepatic fatty acid synthase and malic enzyme activity, compared to CON and PIO groups (Figure 3D). Hepatic carnitine palmitoyltransferase and β-oxidation were not significantly altered by HON; however, PIO significantly increased hepatic carnitine palmitoyltransferase activity and β-oxidation (Figure 3D).

### 2.4. Effect of HON on Inflammation

**Figure 4:**
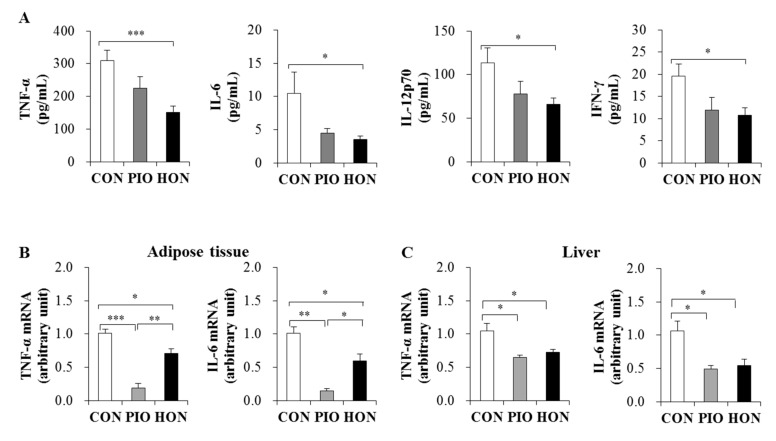
Effect of dietary HON on plasma pro-inflammatory cytokine levels (**A**), and the mRNA expression of pro-inflammatory cytokines in adipose tissue and livers (**B**,**C**) of *db*/*db* mice. Male C57BL/KsJ-*db*/*db* mice were fed a normal diet with or without HON (0.02%, *w*/*w*) or PIO (0.01%, *w*/*w*) for 5 weeks (*n* = 10 per group). Data are presented as mean ± SE. ^*^ *p* < 0.05, ^**^ *p* < 0.01, ^***^ *p* < 0.001. CON; control group, PIO; pioglitazone, HON; honokiol, TNF-α; tumor necrosis factor- α, IL-6; interleukin-6, IL-12p70; interleukin-12p70; IFN-γ; interferon-γ.

The levels of pro-inflammatory cytokines such as tumor necrosis factor (TNF)-α, interleukin (IL)-6, IL-12p70, and interferon (IFN)-γ were significantly lower in the plasma of the HON-treated mice compared to the CON group (Figure 4A). PIO-treated mice showed a significant decrease in plasma IFN-γ levels and decreased plasma TNF-α, IL-6, and IL-12p70 levels compared to the CON group (Figure 4A). Moreover, HON and PIO significantly down-regulated the mRNA expression of TNF-α and IL-6 in adipose tissue and the liver compared to the CON group (Figure 4B,C).

## 3. Discussion

This study investigated the effects of HON on hyperglycemia, insulin resistance, dyslipidemia, hepatic steatosis, and inflammation in type 2 diabetic *db*/*db* mice – obesity-induced diabetic animals with insulin resistance. Supplementing mouse diets with HON for 5 weeks significantly lowered blood HbA1c and plasma insulin levels, and improved glucose intolerance and insulin resistance compared to control *db*/*db* mice. These anti-diabetic properties of HON are likely due to the inhibition of hepatic gluconeogenic enzymes, as well as the loss of body fat. We demonstrated that HON improved inflammation and ameliorated hepatic steatosis by inhibiting the activity of hepatic lipogenic enzymes.

PIO is a thiazolidinedione widely used to treat type 2 diabetes. In obese diabetic *db*/*db* mice, PIO improved hyperglycemia, but induced lipid deposition in adipose tissues [20]. In agreement with this previous study, our study shows that PIO significantly decreases fasting blood glucose and blood HbA1c levels, but significantly increases body weight, fat weight, and plasma leptin levels, compared to control *db*/*db* mice. Despite decreased food intake in the PIO group, the increases in body weight and fat weight probably occur through stimulation of de novo lipogenic enzymes activities and inhibition of carnitine palmitoyltransferase activity in adipose tissue. Another possible mechanism by which PIO increases body weight may be due to a reduction of energy expenditure, although we did not determine this. Lamontagne et al. [21] reported that PIO reduced energy expenditure in Zucker diabetic fatty rats, an animal model of type 2 diabetes. In contrast to PIO, treatment with HON significantly decreased fat weight by inhibiting the activity of adipose fatty acid synthase, a multifunctional enzyme that plays a central role in de novo lipogenesis by catalyzing all the reactions in the conversion of acetyl-CoA and malonyl-CoA to long-chain fatty acids. In addition, HON treatment significantly decreased plasma insulin levels and improved impaired glucose tolerance and insulin resistance. We found that HON significantly decreased the levels of blood HbA1c, a useful biomarker for the long-term monitoring of blood glucose that allows for the analysis of two to three months of cumulative glycemic history [22]. The HbA1c is a simple and reliable marker used to accurately estimate insulin sensitivity in persons with diabetes [23]. Also, in obese subjects with a normal fasting blood glucose level, high levels of HbA1c are suggested as a screening tool to detect insulin resistance or impairment of insulin secretion [24,25]. Therefore, the decreased level of HbA1c observed in HON group may be related to the improvement of insulin resistance and protection of β-cells by HON. As excessive fat accumulation in adipose tissue contributes to insulin resistance, and obesity is accompanied by hyperinsulinemia [23], the anti-adiposity effect of HON may be partly associated with an improvement in insulin signaling and hyperinsulinemia.

In obesity, adipose inflammation as well as increased adiposity is linked to the development of insulin resistance [4,5,6,7]. Adipose tissue-derived pro-inflammatory cytokines such as TNF-α and IL-6 can cause insulin resistance in adipose tissue, liver, and skeletal muscle by impairing insulin action [5,26,27]. Neutralization or knockdown of inflammatory mediators, including TNF-α and IL-6, are protective against insulin resistance in obese rodents [28,29]. However, a recent study showed that blocking IL-6 trans-signaling prevents high-fat diet-induced adipose tissue macrophage recruitment but does not improve insulin resistance [30]. Shimobayashi et al. [6] have suggested that obesity-mediated insulin resistance in mice precedes macrophage accumulation and causes inflammation in adipose tissue. Although the causal relationship between adipose tissue inflammation and insulin resistance is unclear, obese diabetic *db*/*db* mice have increased TNF-α and IL-6 mRNA expression in adipose tissue [31], and PIO decreases the mRNA expression of TNF-α and IL-6 in the adipose tissue of subjects with type 2 diabetes [32]. We observed that the mRNA expression of TNF-α and IL-6 in adipose tissue was down-regulated by both HON and PIO treatment. These findings suggest that decreased expression of these pro-inflammatory cytokines in adipose tissue may be related to the improvement of insulin resistance induced by HON.

Insulin resistance is a pathological condition in which insulin-sensitive tissues including the liver and adipose tissue fail to respond to normal circulating levels of insulin [33]. The liver plays a pivotal role in the maintenance of glucose homeostasis. Hepatic glucose production accounts for approximately 90% of endogenous glucose production [34], and it is crucial for the maintenance of normal glucose homeostasis [35]. The inability of insulin to suppress hepatic glucose production is the primary cause of type 2 diabetes [36]. In type 2 diabetes, hepatic glucose production is higher in the post-absorptive state, and fails to be properly suppressed by insulin, primarily due to the increased rate of gluconeogenesis rather than glycogenolysis [37]. PEPCK and G6Pase are key enzymes that regulate hepatic gluconeogenesis [38,39]. The expression/activity of these enzymes increases in the livers of diabetic humans and rodents, including *db*/*db* mice [40,41]. Overexpression of PEPCK in mice increases hepatic glucose production and impairs glucose tolerance [42]. Conversely, silencing cytosolic PEPCK in the liver improves insulin sensitivity in *db*/*db* mice [41]. In addition, mice with a moderate reduction of hepatic G6Pase activity do not develop age-related insulin resistance or obesity [43]. We show that HON significantly inhibited the mRNA expression and activity of hepatic gluconeogenic enzymes including PEPCK and G6Pase. Taken together, these data suggest that the inhibition of hepatic PEPCK and G6Pase observed in HON-treated *db*/*db* mice may be associated with improved insulin resistance.

Impaired suppression of hepatic glucose production is associated with increased intrahepatic triglyceride accumulation, which is a hallmark of NAFLD [44,45]. Most patients with type 2 diabetes experience NAFLD, and NAFLD patients have a higher risk of insulin resistance compared to healthy controls [46,47]. It is suggested that intrahepatic triglyceride content is a strong predictor of insulin action in the liver, skeletal muscle, and adipose tissues [44,45]. Triglyceride accumulation in the liver depends on free fatty acid delivery to the liver, de novo lipogenesis, and the rate of fatty acid oxidation. As insulin continues to stimulate hepatic fatty acid synthesis, even in an insulin-resistant state, insulin resistance and the resulting hyperinsulinemia increases de novo lipogenesis [48,49]. Several studies have demonstrated that *db*/*db* mice have a higher hepatic triglyceride content and increased expression of lipogenic genes and proteins, including fatty acid synthase, compared to *db*/*+* mice [50,51]. We show that HON significantly decreases the activity of lipogenic fatty acid synthase and malic enzyme in the livers of *db*/*db* mice, suggesting that reduced hepatic triglyceride content in response to HON treatment is due to a decrease in hepatic lipogenesis, and is responsible for the improvement in hepatic steatosis. We also observed that supplementation of mouse diets with HON improved hepatic inflammation, as shown by the decrease in TNF-α and IL-6 expression in the liver. Cai et al. [52] reported that hepatic steatosis results in hepatic inflammation through the production of pro-inflammatory cytokines, which leads to both hepatic and systemic insulin resistance. Therefore, suppression of hepatic inflammation by HON may also ameliorate insulin resistance in *db*/*db* mice.

## 4. Materials and Methods

### 4.1. Experimental Animals

Thirty male 4-week-old C57BL/KsJ-*db*/*db* mice were purchased from Jackson Laboratory (Bar Harbor, ME, USA). After the acclimation period of 1 week, the mice were randomly divided into 3 groups (*n* = 10) and fed the respective experimental diets for 5 weeks: a normal control diet (CON; AIN-76 semisynthetic diet) and a normal control diet with either PIO (0.01%, *w*/*w*, Takeda Pharmaceutical Company, Osaka, Japan) or HON (0.02%, *w*/*w*). HON was isolated from the Magnolia obovata bark according to previously reported methods [53].

All mice were maintained in a controlled environment with a light-dark cycle (12:12 h) and constant temperature (24 °C). They were allowed free access to food and tap water, and food intake and body weight were measured daily and weekly, respectively. At the end of the experimental period, all mice were anesthetized with isoflurane (5 mg/kg body weight, Baxter Healthcare Cooperation, Deerfield, IL, USA) after a 12-h fast, and blood samples were collected from the inferior vena cava to quantify blood and plasma biomarkers. After blood collection, adipose tissue (perirenal, retroperitoneal, mesentery, subcutaneous, and scapular white adipose tissue) and livers were promptly removed, washed with physiological saline, weighed, and frozen and kept at −70 °C, until the analyses of enzyme activity and RNA expression. The pancreas was also removed, rinsed with physiological saline and fixed for immunohistochemistry after 5 weeks of treatment. Studies were performed using protocols for animal studies approved by the Kyungpook National University Ethics Committee (Approval No. KNU-2011-49, 18 September 2011).

### 4.2. Blood and Plasma Parameters

The concentration of blood glucose was monitored in the venous blood drawn from the tail vein, using a glucometer (Gluco Dr SuperSensor, Allmedicus, Korea) every week after a 12-h fast. The IPGTT was performed on week 4. After a 12-h fast, mice were injected intraperitoneally with glucose (0.5 g/kg body weight). Blood glucose levels were determined using blood obtained from the tail vein at 0, 30, 60, and 120 min after glucose injection. At the end of the experimental period, blood HbA1c levels were measured using an analyzer (Micromat™ I Hemoglobin A1c Test, Bio-Rad, Hercules, CA, USA). The HOMA-IR was calculated as follows: HOMA-IR = [fasting glucose (mmol/L) × fasting insulin (µL U/mL)]/22.51. The levels of insulin, glucagon, leptin, and cytokines (TNF-α, IL-6, IL12p70, and IFN-γ) were measured in the plasma, using a multiplex detection kit obtained from Bio-Rad Laboratories (Hercules, CA, USA). All samples were assayed in duplicate and analyzed with a Luminex 200 Labmap system (Luminex, Austin, TX, USA) and Bio-Plex Manager software version 4.1.1 (Bio-Rad, Hercules, CA, USA).

### 4.3. Plasma and Hepatic Lipids

Plasma lipid concentrations were determined using commercially available kits. Free fatty acids levels were measured using the Wako enzymatic kit (Wako Chemicals, Richmond, VA, USA), and triglyceride, total cholesterol, and HDL-cholesterol levels were measured using Asan enzymatic kits (Asan Pharm. Co., Ltd., Seoul, Korea). The atherogenic index was calculated from lipid profiles as follows: atherogenic index = [(total cholesterol) − (HDL-cholesterol)]/(HDL-cholesterol). Hepatic lipids were extracted using the method described by Folch et al. [54], and hepatic lipid levels were analyzed using the enzymatic kits used for plasma analyses.

### 4.4. Hepatic and Adipose Tissue Enzymatic Activity

To measure the activity of glucose- and/or lipid-regulating enzymes in the liver and epididymal adipose tissue, samples were prepared and analyzed as previously described [55]. PEPCK activity was determined according to the method described by Bentle and Lardy [56], with slight modifications. The reaction mixture contained the following in a 1 mL final volume: 50 mM sodium HEPES (pH 6.5), 1 mM IDP, 1 mM MnCl2, 1 mM dithiothreitol, 0.25 mM NADH, 2 mM phosphoenolpyruvate, 50 mM NaHCO_3_, 7.2 units of malic dehydrogenase and hepatic cytosol. The activity of G6Pase was measured in the microsome using a spectrophotometric assay according to the protocol described by Alegre et al. [57], with slight modifications, and containing 100 mmol/L sodium HEPES (pH 6.5), 26.5 mmol/L glucose-6-phosphate, 1.8 mmol/L EDTA, both previously adjusted to pH 6.5, 2 mmol/L NADP^+^, 0.6 kIU/L mutarotase, and 6 kIU/L glucose dehydrogenase. Glucokinase activity was determined in the hepatic cytosol using a spectrophotometric assay described by Davidson and Arion [58], with slight modifications. Briefly, the oxidation of glucose-6-phosphate at 37 °C was catalyzed by glucose-6-phosphate dehydrogenase, with NAD^+^ as the electron acceptor. Fatty acid synthase activity was determined using the protocol described by Nepokroeff et al. [59], by monitoring the malonyl-CoA-dependent oxidation of NADPH at 340 nm (nmol/min/mg protein), as a measure of fatty acid synthase activity. The activity of malic enzyme was determined as previously described by Ochoa [60]. The cytosolic enzyme was mixed with 0.2 mM triethanolamine buffer (pH 7.4), 1.5 mM L-malate, 12 mM MnCl_2_, and 680 M NADP^+^, and analyzed for 1 min at 340 nm (26 °C) on a spectrophotometer. Carnitine palmitoyltransferase activity was determined according to the method published by Markwell et al. [61], and the results were expressed as nmol/min/mg protein. Fatty acid β-oxidation activity was measured spectrophotometrically by monitoring the reduction of NAD to NADH in the presence of palmitoyl-CoA, as described by Lazarow [62]. The amount of protein in each enzyme source was determined using the Bradford method [63], using bovine serum albumin to generate the standard curve.

### 4.5. Hepatic Glycogen Assay

The concentration of hepatic glycogen was determined as previously described by Seifter et al. [64], with modifications. Briefly, liver tissue was homogenized in 5 volumes of a 30% (*w*/*v*) KOH solution, and dissolved at 100 °C for 30 min. Glycogen levels were determined by treatment with an anthrone reagent (2 g anthrone/L of 95% (*v*/*v*) H_2_SO_4_) and measuring the absorbance at 620 nm.

### 4.6. RNA Extraction and Analysis of Gene Expression

The liver and epididymal adipose tissues were homogenized in TRIzol reagent (Invitrogen Life Technologies, Grand Island, NY, USA) and total RNA was extracted according to the manufacturer’s instructions. DNase digestion was used for any contaminating DNA, and the RNA was washed with ethanol and re-precipitated with isopropanol to remove any contaminating phenol. For quality control, RNA purity and integrity were evaluated using the Agilent 2100 Bioanalyzer (Agilent Technologies, Palo Alto, CA, USA). Total RNA (1 μg) was reverse-transcribed to synthesize cDNA using the QuantiTect^®^ reverse transcription kit (Qiagen, Hilden, North Rhine-Westphalia, Germany). mRNA expression was quantified by quantitative reverse transcription PCR (RT-qPCR), using the SYBR green PCR kit (Qiagen, Hilden, North Rhine-Westphalia, Germany), and the CFX96TM real-time system (Bio-Rad, Hercules, CA, USA). Cycle thresholds (Ct) were determined based on the SYBR green emission intensity during the exponential phase. Using the 2^−ΔΔ*C*t^ method, the fold changes were calculated; transcripts of GAPDH were also amplified from the samples and used as the housekeeping gene [65].

### 4.7. Morphological Analysis of the Liver and Immunohistochemical Analysis of the Pancreas

After 5 weeks of treatment, the liver was removed and fixed in a buffer solution containing 10% formalin. Fixed tissues were processed routinely for paraffin embedding, and 4-μm sections were prepared and dyed with hematoxylin-eosin (H&E). The pancreas was fixed in a buffer solution containing 10% formalin and embedded in paraffin for immunohistochemical staining of insulin. Anti-insulin primary antibody (Santa Cruz Biotech, Inc., Santa Cruz, CA, USA) was used. Stained areas were viewed using an optical microscope with a magnifying power of ×200.

### 4.8. Statistical Analysis

All data are presented as the mean ± S.E. The data were evaluated by one-way ANOVA using the SPSS program, and by determining the differences between the means with Tukey’s post-hoc test. Values were considered statistically significant at *p* < 0.05.

## 5. Conclusions

We have shown that HON reduced excessive adiposity, hepatic steatosis, and insulin resistance in type 2 diabetic *db*/*db* mice. The anti-adiposity and anti-hepatic steatosis effects of HON are partly attributable to its ability to decrease lipogenesis in adipose tissue and the liver, which is associated with improving insulin resistance. HON also has anti-inflammatory properties via the inhibition of pro-inflammatory cytokine expression in adipose tissue and the liver, which may contribute to enhanced insulin sensitivity.

## Abbreviations

G6Pase	Glucose-6-phosphatase
HbA1c	Gycosylated hemoglobin
HDL	High-density lipoprotein
HOMA-IR	Homeostatic model assessment index of insulin resistance
HON	Honokiol
IFN	Interferon
IL	Interleukin
IPGTT	Intraperitoneal glucose tolerance test
NAFLD	Nonalcoholic fatty liver disease
PEPCK	Phosphoenolpyruvate carboxykinase
PIO	Pioglitazone
TNF-α	Tumor necrosis factor-α
